# Advancing user-centric design and technology adoption for aging populations: a multifaceted approach

**DOI:** 10.3389/fpubh.2024.1469815

**Published:** 2024-12-06

**Authors:** Andreea Stamate, Mircea-Dan Marzan, Magdalena Velciu, Cosmina Paul, Luiza Spiru

**Affiliations:** ^1^Research Department, Ana Aslan International Foundation, Bucharest, Romania; ^2^Department of Geriatrics, Gerontology, and Old Age Psychiatry and Longevity Medicine, Carol Davila University of Medicine and Pharmacy, Bucharest, Romania

**Keywords:** older adults, digital devices, usability, best practices, interdisciplinary communication

## Abstract

The global demographic shift toward an aging population necessitates a nuanced approach to developing and adopting assistive technologies tailored for older adults. This paper synthesizes key challenges, strategies, and recommendations identified in addressing the complex landscape of technology adoption and usage among aging populations. User-centric design and co-creation initiatives are vital for developing assistive technologies that meet the needs of older adults. These initiatives involve engaging older adults in activities like workshops, focus groups, and design sessions to gather feedback and refine technology solutions, ensuring they are accessible, intuitive, and effective. Challenges such as participant selection, cultural attitudes, and trust-building mechanisms are paramount in ensuring meaningful user involvement in technology development processes. Accurate assessment of older adults’ technological literacy is identified as critical for designing and implementing digital solutions. The unreliability of self-reported proficiency necessitates objective measures in assessments to counter potential biases and ensure accurate insights into user capabilities. The fragmented digital ecosystem and resulting digital divide among older adults pose significant barriers to technology adoption and usage. The role of caregivers in technology acceptance highlights the need for integrated models that encompass the caregiver perspective, reducing adoption barriers and fostering meaningful engagement with assistive technologies. Interdisciplinary collaboration and robust research standards are essential in advancing technology adoption and addressing societal inequalities. Prioritizing user-centric design, integrating caregivers into technology adoption models, and fostering collaborative efforts across disciplines can significantly improve technology acceptance and enhance the quality of life for older adults in an increasingly digital era.

## 1 Background

The concept of Healthy and Independent Living at Home has surged in relevance due to aging populations, rising healthcare costs, technological advancements, and the impact of events like the COVID-19 pandemic. It emphasizes the importance of empowering individuals to maintain their well-being and autonomy within their own homes through proactive health management and the adoption of supportive technologies. There are many projects, organizations, and networks aiming to bring progress in the field of health literacy, digital health, age-friendly environments, integrated care, etc. Still, just a few of the products that are being designed for older adults are currently on the market, and even fewer gain people’s acceptance and adoption (1). A significant number of older adults are still unable to initiate healthcare technology on their own without help (2).

Several factors can contribute to the failure of digital products, some of these relate to: (1) Lack of User-Centric Design—many digital products fail because they do not adequately consider the needs, preferences, and capabilities of older adults. User interface designs may be complex, cluttered, or difficult to navigate, making them inaccessible or frustrating for older users (3); (2) Underestimation or overestimation of Technological Literacy—there is large heterogeneity in digital skills among older users (4), and more importantly they are not always capable of assessing themselves accurately; (3) Fragmented Ecosystem—the digital landscape for older adults is often fragmented, with a multitude of products and services targeting various aspects of aging sometimes with measurement issues (5–7); (4) Lack of Integrated Technology Adoption Models—assistive technologies overlook the crucial role of caregivers in supporting and influencing older adults acceptance, contributing to digital divides and failures in technology development and adoption (8); (5) Ignoring the social innovation dimension—neglecting social innovation in assistive technologies widens the digital gap, highlighting a need for joint government and businesses effort to bridge these disparities; (6) Lack of systematized literature—Despite a growing focus on older adults’ technology use, standardized literature and research practices are lacking, highlighting the need for interdisciplinary collaboration to establish standards. This fragmentation can make it difficult for older adults to find and adopt products that meet their needs, leading to a lack of uptake and eventual failure for many offerings.

In this article we will elaborate on these factors, that we identified through experience, from our observation while working with older adults, as well as the extant literature. A means to reduce some of these failings would be by sharing knowledge, enabling us to learn from past mistakes, address common challenges, and incorporate best practices into their designs. By collaborating and sharing expertise, developers can create more accessible, user-friendly technologies that better meet the needs and preferences of older adults. This collaborative approach fosters innovation and empowers older adults by ensuring that digital solutions are designed with their unique requirements in mind, ultimately increasing the likelihood of success and adoption in this demographic.

The development of health technologies for older adults has evolved significantly since the 1960s, starting with electronic medical records (9). The 1970s brought advancements in medical imaging like CT scans and MRIs, revolutionizing diagnostics. In the 2000s, consumer-grade devices such as Fitbit and mobile health apps empowered individuals in proactive health management (10). The COVID-19 pandemic further accelerated telehealth adoption, highlighting its role in care continuity (11).

Despite the promise of AI, big data, and digital therapeutics, challenges remain in ensuring that older adults can fully benefit from these technologies. Addressing these challenges requires adaptable, user-friendly, and equitable designs, which emphasizes the importance of engaging older adults in the co-design process. Efforts like the Horizon 2020 program have sought to address similar societal challenges by promoting collaboration between academia, entrepreneurship, and innovation, although more work is needed to bridge the gap between scientific advancements and societal needs (12, 13).

Building on these collaborative efforts, AAIF has actively contributed to advancing digital health through participation in 30 projects over the past 18 years, focusing specifically on e-Health and digital solutions for older populations. By sharing our experiences, we demonstrate how our contributions to product design, alongside collaborations with medical, technology, and business entities, have enhanced the quality of life for older adults. Our role as an end-user partner continues to be crucial in supporting the development of innovative and user-centered solutions for this demographic.

Our experience has shown the critical importance of involving older adults in the design process of applications targeting their needs. The acceptance of a product is heavily dependent on the user experience it provides (14). Moreover, recent research has expanded the focus on technology acceptance by exploring the multi-stage process of technology acceptability and adoption. In this framework, acceptability refers to an individual’s perception of a system prior to use, while adoption signifies the attainment of sustained use (15). To ensure diverse input, we conduct tests of digital products with partners across the EU, often encountering differing opinions based on the pilot country. Analyzing these results requires understanding the underlying reasons for this variability, which can provide valuable insights into tailoring solutions to different cultural and contextual needs. Hence, in line with the recent literature, we emphasize the role played by culture more than demographics (16, 17).

## 2 Lack of user-centric design

The projected global proportion of older individuals is expected to reach approximately 16% by 2050, indicating that one out of every six individuals worldwide will be over the age of 65. Additionally, the population of those over 80 years old appears to be increasing at an accelerated pace (18). This further emphasizes the importance of research that involves older adults as co-designers, however, co-creation in itself presents with several challenges: (1) Do those people who participate in a co-creation project represent the target audience adequately?; (2) Do people from different countries provide different opinions on the tested product, and why?

The diversity of user needs, as emphasized by Von Hippel et al. (19), underscores the significance of careful participant selection in participatory product design. This ensures that co-creation initiatives generate ideas that cater to the expectations of a broad spectrum of consumers. Companies stand to gain valuable insights from these co-creating consumers, as noted by Mahr et al. (20). Additionally, participants’ willingness to engage in creative sessions is often influenced by specific personality traits or interests related to the co-creation topic or product category. In co-creation endeavors, including those involving older adults, the diversity of participants holds greater importance than the representativeness of the consumer sample. However, it is essential that participants derive meaningful experiences and can provide relevant contributions.

User-centric studies emphasize the importance of considering both the diversity of participants and the technological and psychological aspects of user interaction. Beyond developing technologies to manage stress, it is vital to address users’ psychological responses. Research shows that reducing stress can greatly enhance user experience. Yagil et al. (21) found that older adults’ coping strategies significantly influenced their stress levels and well-being, while Strutt et al. (22) highlighted the impact of stress and coping mechanisms during times like the COVID-19 pandemic. Performance anxiety can also hinder technology use, especially during testing. Seamlessly integrating evaluation systems into daily life can yield more accurate results, underscoring the need for adaptable and user-friendly designs to meet the diverse needs of older adults and promote better engagement.

### 2.1 The individual characteristics bias

Older adults are a heterogeneous group with diverse socio-cultural or educational backgrounds, ICTs skill, as well as varying experiences with health technologies. These factors influence their knowledge, understanding and perception of technology, ultimately affecting how they provide feedback during co-creation.

Technology adoption among older adults is often hindered by stereotypes and a lack of understanding of their specific needs (23). This disconnect is partly because researchers and developers are typically not from this demographic (24). Current practices often overlook critical factors like motivation, diversity within the group, and the contexts of technology use.

Effective use of assistive technology requires not only technical skills but also considerations related to design, familiarity, and the physical and social environment. Predictors of technology use include sociodemographic factors, attitudes, and cognitive abilities (25, 26). While decreased cognitive capacity impacts daily activities, cognitive ability alone does not fully explain performance variance (27). Malinowsky et al. (28) identified three factors affecting older adults’ ability to manage electronic technology: intrapersonal capacities (e.g., stress management, attention, and recall), environmental characteristics, and the interaction between these variables. These findings emphasize that variability in personal capacities, rather than overall cognitive status, significantly affects technology management, supporting the concept of person-environment fit (29).

Lee and Coughlin (24) identified 10 factors influencing older adults’ technology adoption, including value, usability, affordability, and social support. These factors go beyond traditional models like the Technology Acceptance Model, suggesting a need to consider individual and social characteristics for better adoption. Additional influences like interoperability and system reliability point to gaps in current understanding, requiring more comprehensive research.

Predictors of ICT adoption include behavioral factors, demographics, and individual differences (30, 31). Chopik et al. (32) found that traits like need for cognition and optimism positively predicted ICT use, while cynical hostility was a negative predictor. These differences influence attitudes, perceived benefits, and the use of ICT for health and social engagement, showing the complexity of individual pathways to adoption.

In conclusion, understanding technology adoption among older adults requires a multifaceted approach beyond traditional models, incorporating cognitive, demographic, and individual factors, along with personal capacities and environmental contexts. Addressing these diverse elements can lead to more effective, tailored technology designs and support mechanisms, fostering greater adoption and use among older adults.

### 2.2 Differences in sharing their opinions and giving feedback

Older adults have vastly different life experiences. For example, those aged 65 and above residing in Eastern European countries have lived through prolonged periods under authoritarian regimes, which may lead to reluctance in sharing critical opinions. In former communist countries like Romania, individuals may be less inclined to voice dissent due to past restrictions on freedom of expression. This background can challenge co-creation processes by limiting honest input and open dialogue, potentially overlooking innovations. Therefore, it is crucial to foster critical thinking and motivate older adults to express their authentic opinions, especially when assessing the quality of services, they need.

In contrast, individuals in Western cultures are more accustomed to providing critical feedback and openly expressing their opinions, even if they contradict prevailing views. This openness fosters a dynamic exchange of ideas, encouraging diverse perspectives and innovative solutions during co-creation initiatives, leading to richer discussions and more effective outcomes.

In our organization we strive to create an environment of trust through several key strategies. First, we ensure transparent communication by clearly outlining the goals of the co-creation process and emphasizing the importance of honest feedback. Participants are informed that their insights will directly influence the final product. To further encourage feedback, we actively invite participants to share their thoughts on specific aspects of the prototype by asking open-ended questions, making it clear that all opinions are valued. We also acknowledge contributions by recognizing and appreciating participants’ input, highlighting how their feedback has led to tangible improvements in design or functionality. Creating a safe space is another critical strategy. We foster a non-judgmental atmosphere where participants feel comfortable expressing dissenting views, reassuring them that their honesty will not lead to negative repercussions. Building relationships is equally important; we take time to engage in informal conversations, helping to create a sense of community and shared purpose. Finally, we provide support throughout the process, ensuring that participants feel confident in sharing their perspectives by offering additional resources or assistance if they encounter difficulties. By implementing these strategies, we aim to cultivate a trusting environment where participants feel empowered to contribute openly and authentically.

### 2.3 Differences in willingness to explore new things, healthcare technology, and continuous learning

Technology is rapidly transforming healthcare. The prevention of disease is the new cornerstone of healthy aging, encompassing the promotion of a healthy lifestyle, early detection of physical and cognitive deterioration, and personalized intervention. Social networking and lifelong learning are essential components of this approach.

The variance in older adults’ willingness to explore new healthcare technology and engage in continuous learning significantly impacts their adoption and utilization of innovative solutions. Some older adults exhibit a strong curiosity and openness to new experiences, eagerly embracing emerging technologies and actively seeking lifelong learning opportunities. These individuals readily adapt to new healthcare technologies, exploring their benefits and incorporating them into their daily routines. Their enthusiasm enables them to stay informed about the latest advancements and navigate digital platforms confidently to manage their health.

Conversely, some older adults may display cautious attitude toward new technologies, preferring familiar routines and traditional healthcare approaches. This reluctance may stem from concerns about complexity, usability, or perceived lack of relevance to their health needs. Additionally, older adults with limited access to digital resources or lower digital literacy levels may face barriers to engaging with healthcare technology effectively.

To effectively address the diverse willingness of older adults to engage with healthcare technology in co-creation processes, it is essential to tailor outreach efforts, offer flexible participation options, provide personalized support and training. By segmenting participants based on their comfort with technology, offering various engagement channels, and maintaining open communication, co-creation initiatives can ensure inclusive and meaningful involvement of older adults in shaping healthcare technologies.

Nevertheless, success is not assured, as it involves a complex, multi-task, and multi-stakeholder process that surpasses traditional citizen participation efforts (33). Given the inherent openness and complexity of co-creation, establishing strict guidelines proves challenging. However, the insights gleaned through knowledge transfer can offer valuable evidence on methods to effectively co-create more user-centric public services with and for older adults.

## 3 Underestimation or overestimation of technological literacy

As previously noted, a crucial consideration in designing new applications and facilitating co-creation engagement is evaluating the computer proficiency of older adults. Addressing the unique requirements and limitations of older individuals when crafting digital technologies can enhance user experience. This focus can drive advancements in assessment and diagnosis, with the goal of optimizing patient advantages while minimizing associated risks (34).

Proficiency in computer and internet skills significantly influences technology adoption, with advanced usage, positive attitudes, and self-efficacy closely linked. However, there is a notable lack of inclusive research focused on older adults, as current assessments often overlook diverse user abilities. Deficiencies in computer skills can create barriers to embracing new technologies, with research indicating that higher proficiency correlates with better technology adoption (35, 36) and increased self-efficacy (37). Conversely, a lack of proficiency can heighten computer anxiety (38, 39).

While many studies focus on younger generations, research and design for older adults remain insufficient (40). Effective assessment of digital competencies is essential for helping older adults leverage digital healthcare. In our recent study (currently under review), we compared self-reported and objective digital skills assessments using the Digital Foundation Skills framework. Data from 51 older adults in Romania revealed significant discrepancies in areas such as Digital foundation skills, while self-assessments were more accurate in Handling information and content. These results underscore the need for objective measures to evaluate digital competence comprehensively.

Recent research employs various instruments for measuring digital literacy, with self-reported measures like the eHealth Literacy Scale (40) often failing to reflect actual competencies. In contrast, objective assessments utilize structured tasks to gage real skills, highlighting the necessity for reliable evaluation tools in assessing digital literacy among older adults.

Awareness of older adults’ computer proficiency is essential when designing new applications. Addressing the unique needs and limitations of older adults in the design of digital technologies can ensure a better user experience. Consequently, forthcoming studies should consider incorporating objective measures when assessing digital competence as a variable. Furthermore, understanding older adults’ digital skills is critical, especially regarding digital cognitive testing. One challenge in computerized cognitive assessment is that varying levels of computer experience can influence test performance, regardless of cognitive ability. Relying solely on computerized tests for clinical decisions may lead to overdiagnosis in individuals with limited computer experience and under diagnosis in those with extensive computer skills (41). This consideration has the potential to advance assessment and diagnosis technologies, aiming to maximize patient benefits while minimizing potential risks (34).

## 4 Fragmented ecosystem

The fragmented ecosystem in the digital landscape for older adults exacerbates the challenge of ensuring the accuracy of measurement in digital devices. With numerous products and services targeting different aspects of aging, older adults face difficulty in finding and adopting solutions that truly meet their needs. This fragmentation not only hampers uptake but also contributes to the failure of many offerings. Furthermore, it becomes crucial to ensure that digital devices that are being marketed provide accurate measurements, as unreliable or inconsistent data further compounds the already complex task of navigating the fragmented digital landscape for older adults. Accurate measurement enhances the trustworthiness of digital solutions, increasing their likelihood of adoption and success amidst the challenges posed by the fragmented ecosystem.

Recent research highlights significant issues with the usability and reliability of digital health tools for older adults. Wearable devices are increasingly used in health for biomedical research and clinical care, promoting personalized and preventive medicine. While they offer many benefits, wearables also pose risks related to privacy and data sharing. The literature has often treated technical and ethical issues separately and only partially explored their role in biomedical knowledge. Canali et al. (5) have provided an overview of wearables’ functions in monitoring, screening, detection, and prediction and identified four concerns: data quality, balanced estimations, health equity, and fairness. To address these, the authors recommend focusing on local standards of quality, interoperability, access, and representatively.

Another issues is the lack of interoperability, which is recognized as a major obstacle to the adoption and implementation of digital health technologies and the overall digital transformation of healthcare OECD (42). Overcoming this challenge demands awareness and collaboration among all stakeholders, beginning with a common understanding of digital health standards.

Many devices struggle with usability and accuracy, affecting their effectiveness (6). For example, David Conroy, a Penn State kinesiology professor, highlights that current physical activity trackers fail to accurately monitor older adults, as wrist-worn devices can misinterpret arm movements and skew step counts. Wagner and Ogawa (7) emphasizes that, due to older adults’ slower walking pace, current trackers struggle with subtler movement signals, and advocates for developing technology that can differentiate activities specific to their daily routines to enhance health monitoring and promote healthy aging. The reliability of health-monitoring devices is crucial for effective health management. As part of our activity, we have recently assessed the reliability of a new tool that enables easy, and remote assessment of hand grip strength (43). The device we evaluated, Squegg Smart Grip Trainer, is a practical, efficient, and affordable instrument. The Squegg® Smart Dynamometer and Hand Grip Trainer is a digital, handheld device that functions as a dynamometer. It facilitates Bluetooth connectivity to a mobile device (such as a phone or tablet) to visually display and record handgrip force data during rapid grip and release movements over time in a cloud-based system. To assess its reliability, we compared participants measurements on the gold standard dynamometer, the Jamar dynamometer to the measurements obtained with the Squegg. Participants’ maximal grip strength for both their dominant and nondominant hands was measured with both devices. We used ICCs to test interinstrument reliability. The overall ICC value was computed across all data collectively, resulting in an overall ICC of 0.912, which indicates a good to excellent interinstrument agreement between the two devices.

Digital solutions for assessment and management, developed by AAL consortia, are often intended for use alongside various wearables. These wearables feature sensory and audio-visual technology, allowing for the simultaneous collection of primary and secondary data (44). This capability enables the storage of valuable behavioral data (45). Although wearable devices offer numerous benefits for monitoring and intervening in the health conditions of older adults, they also present new challenges. One challenge relates to the proper evaluation of digital devices. Have devices undergone testing which establishes that they are reliable measure of the construct under investigation?

Our own experience does not suggest that they always do. When selecting devices for training or monitoring, it is crucial to ensure they have been specifically tested for the intended purpose as well as having been tested on the population its intended for.

The fragmentation of electronic Health Information Systems, due to the independent development of diverse ICT tools and methods, hampers the seamless exchange of patient information and contributes to medical errors (46). To address these issues, it is crucial to adopt standardized healthcare terminology, improve education strategies, design user-friendly interfaces, ensure privacy and security, and connect legacy systems to the health network for achieving complete interoperability (46).

Ensuring device reliability involves rigorous testing against gold standards to validate accuracy and integrating real-world user feedback to refine performance. Transparency in quality standards is crucial to building user trust and ensuring that devices meet the needs of older adults consistently. Addressing these issues through standardization, user-centric design, comprehensive testing, and transparent communication can mitigate the challenges posed by fragmentation and enhance the effectiveness of digital solutions for older adults (47).

## 5 Lack of integrated technology adoption models

### 5.1 Lack of technology adoption models for older adults

Technology Adoption Models became one of the most widely cited theory in the information system literature. Though, its adaptation in the technology adoption literature and practice in the health and care sector started just recently.

The Technology Adoption Model (48), often referred as TAM, and developed in the context of the adult working population, went unchanged for decades. The maturation of the discourse started in the 2000s: developed from the TAM, The Unified Theory of Acceptance and Use of the Technology (UTAUT, (49)) has become widely implemented (50). UTAUT has been constantly improved as it introduced new contextual moderating variables, increasing its reliability (see UTAUT2 and UTAUT3).

The development of theoretical models addressing technology acceptance behavior among older adults began just a decade ago (51, 52). More recently, Wutz et al. (53) adapted the Unified Theory of Acceptance and Use of Technology (UTAUT) to the healthcare sector, highlighting the unique characteristics of the sector and the distinct market segmentation within the post-working population.

### 5.2 Lack of integrated technology adoption models

The usage of the assistive technology by older adults requires the support of the formal or informal caregivers or their family. This support may take various forms, more often implying the acquisition of the technology and technical support - to installing and providing basic tech aid (such as, re-establishing an interrupted Wi-Fi connection, plug and play, setups, etc.). In fact, this support acts as an intergenerational linkage by introducing the older adult to what is newly advanced in the market. Moreover, informal caregivers contribute in various ways to the adoption of technology by older adults, from passing to them their old technological products, to being part of the technological solution, such as responding to alarms or interpreting health or well-being data.

While the role of caregivers in technology adoption is crucial, scholars and practitioners in assistive technologies do not equally integrate stakeholders, thereby failing to adequately account for their acceptability. A more comprehensive approach is necessary to advance Technology Adoption Models, which should move beyond the traditional focus solely on older adults as users and also consider the critical role of caregivers in the acceptance and adoption of products and services.

The failure to develop, test, and advance Technology Adoption Models that integrate all stakeholders leads to two significant consequences: (1) the unnoticed escalation of caregivers’ physical and psychological burdens in promoting and supporting new products, and (2) the perpetuation of digital divides and shortcomings in technology development and adoption among the older adult population. Therefore, a more comprehensive approach is necessary to advance Technology Adoption Models, which should move beyond the traditional focus solely on older adults as users and consider the critical role of caregivers in the acceptance and adoption of products and services.

Studies have shown that technical support from family members and social influence significantly impact older adults’ acceptance of assistive technologies. Accordingly, Technology Adoption Models developed for this demographic incorporate these variables, among others (54, 55). In some models, the caregiver’s role is subsumed under the Social Influence variable (56), while others treat it as distinct (53). As caregivers serve as key facilitators of technology adoption, the gap in understanding how they justify and structure their acceptance of new products for older adults is closing.

## 6 Ignoring the social innovation dimension

The health and care sectors are facing immense pressure due to societal challenges, including aging populations, a shrinking workforce, and increasing demands on the medical field. While governments may view these demographic shifts as a financial strain, businesses are increasingly recognizing the growing “gray market” as a promising opportunity. Despite this potential, adoption rates of new technologies in the care sector remain low (57), as technological innovation cannot progress without parallel advancements in social innovation. Social innovation encompasses the creation of new job roles, specialized training programs, and flexible enrollment practices. These developments are crucial for integrating emerging positions—such as Digital Health Specialists, Community Health Workers, Data Analysts, Health Informaticians, and Patient Advocates—into the healthcare system. Additionally, all roles within the care sector must embrace assistive technology practices to cultivate a culture of tech-enhanced care.

Adhering to best practices, such as establishing validation bodies similar to Germany’s DIGA (Digitale Gesundheitsanwendungen), would help ensure the effectiveness, safety, and user-friendliness of assistive technologies (58). Furthermore, policy recommendations should emphasize the incorporation of horizontal principles, particularly gender equality, in the development of health and care technologies. Although women constitute the majority of the global healthcare workforce, they are significantly underrepresented in leadership positions and technological innovation. Addressing these disparities is essential for achieving more equitable progress (59).

## 7 Lack of systematized literature

Since the advent of the digital revolution, scholars and professionals have increasingly explored and documented the knowledge, attitudes, and behaviors of older adults as end-users of assistive technologies. Though, a dearth of organized literature and research protocols persist, acceptability of technology is still insufficiently understood and the adoption rates stay low (15, 60). We will refer in the following to four instances: (1) unclarified terminology, (2) insufficient practice standardization, (3) inefficient market segmentation, (4) not accounting for cultural factors.

Systematic literature reviews show that ambiguity in the healthcare literature persists and the terminology is still unclarified. For instance, based on a systematic review of the usage of acceptance, acceptability, and adoption, Nadal et al. (15) argue that many studies give their own definitions and do not theorize or define acceptability. Moreover, researchers would also prefer to build their own surveys, and no standardized tools exist, which inhibit the comparability of results.

The heterogeneous nature of the older adult demographic has prompted the development of gerontographic segmentation (61–63) and technology adoption models (TAM) specifically tailored for this population (64, 65), though most of the technology developers would still refer only to age (i.e., 55+ or 65+) as an inclusion criteria (66) and continue to see older adults as a homogeneous population.

Moreover, within the same population of projects dedicated to technology development (66), cross-country studies do not account for country or regional cultural differences, while literature in this domain would point to how culture affect technology adoption (67, 68).

## 8 Conclusion

In conclusion, addressing the challenges faced by aging populations in adopting and benefiting from assistive technologies requires a multi-faceted approach. To enhance the effectiveness of these technologies, several actionable steps should be considered: (1) Enhance User-Centric Design: Engage older adults directly in the design process through co-creation initiatives. This should include diverse participant selection, promoting cultural sensitivity in feedback collection, and fostering open communication to ensure technologies are tailored to the unique needs of this demographic. Future research should explore the most effective methods for involving older adults in these processes, examine the long-term impacts of their involvement on technology adoption, and investigate how different co-creation models can be adapted across cultural contexts; (2) Improve Assessment of Technological Literacy: Develop and implement standardized tools to accurately assess the technological literacy of older adults. Future research should focus on creating comprehensive frameworks that consider varying levels of digital proficiency and how these differences impact the use and adoption of assistive technologies. Additionally, research should examine the efficacy of tailored digital literacy training programs and investigate the relationship between digital literacy improvement and increased technology adoption and satisfaction among older adults; (3) Integrate Caregivers into Technology Adoption Models: Caregivers play a crucial role in supporting older adults in using new technologies. Future research should investigate strategies to involve caregivers more systematically in the adoption process, focusing on how their support can enhance user experience and technology acceptance among older adults. Research should also explore the development of training programs for caregivers to better equip them to assist older adults with technology use and identify the barriers caregivers face in this role; (4) Address Economic and Digital Disparities: To narrow the digital gap among older adults, it is essential to address economic barriers and improve digital literacy. Policy initiatives should aim to provide affordable access to technology and offer digital education programs tailored to older adults. Future research should explore the long-term effects of such interventions on technology adoption and the overall quality of life for older adults, as well as the effectiveness of different types of digital literacy programs in various socio-economic settings; (5) Develop Organized Literature and Standards: The fragmented digital ecosystem calls for a unified effort to create organized literature and establish clear standards for understanding older adults as users of assistive technologies. Future research should focus on developing interdisciplinary guidelines that facilitate better communication and collaboration across fields such as gerontology, human-computer interaction, and technology development. Additionally, research should aim to create a comprehensive taxonomy of assistive technologies that accounts for varying needs and capabilities among older adults; (6) Promote Collaborative Efforts Across Disciplines: To advance technology adoption for aging populations, collaborative efforts across various disciplines are imperative. Future research should examine models of cross-disciplinary collaboration that have successfully improved technology adoption and adapt these models to the context of assistive technologies for older adults. Further studies should also investigate the impact of multi-disciplinary research on innovation and the effectiveness of assistive technologies; (7) Explore Ethical and Privacy Concerns: As assistive technologies become more advanced and integrated into the daily lives of older adults, ethical and privacy concerns become increasingly important. Future research should focus on understanding the privacy concerns of older adults and developing guidelines that ensure these technologies are used ethically and with respect for user privacy. Additionally, studies should explore how different demographic groups within older populations perceive these concerns and the best ways to address them; (8) Examine Longitudinal Impacts of Technology Use: Future research should investigate the long-term effects of assistive technology use on the mental, emotional, and physical well-being of older adults. This includes studying the potential for technology to mitigate feelings of isolation, improve mental health, and enhance overall life satisfaction. Longitudinal studies should also explore how continued use of these technologies influences the independence and daily functioning of older adults over time.

By implementing these actionable steps and focusing on these research areas, we can enhance the design, adoption, and effectiveness of assistive technologies for aging populations. This will ultimately mitigatesocietal inequalities and improve the quality of life for older adults.
